# Initial psychometric validation of the questionnaire on pain caused by spasticity (QPS)

**DOI:** 10.1186/s12955-017-0804-8

**Published:** 2017-11-28

**Authors:** Thorin L. Geister, Donald M. Bushnell, Jie Yang, Yuqiong Zhang, Mona L. Martin, Alev Heilbronn, Zhenhuan Liu

**Affiliations:** 1 Merz Pharmaceuticals GmbH, Eckenheimer Landstraße 100, 60318 Frankfurt, Germany; 2 Health Research Associates, Inc., 6505 216th Street SW, Suite 105, Mountlake Terrace, Seattle, WA 98043 USA; 3 XiangYaBoAi Rehabilitation Hospital, Wanjiali North Road No. 61, Changsha City, Hunan China; 4 MCH Hospital of Dongguan, Children Rehabilitation, 23 YnNeDongErLu, Guancheng District, Dongguan, Guangdong China; 5 Department: Nanhai Affiliated Maternity and Children’s Hospital, Guangzhou University of Traditional Chinese Medicine, 12 Gui Ping Xi Road, Gui Cheng, Foshan, Guangdong China

**Keywords:** Spasticity-related pain, Cerebral palsy, Patient reported outcome measures, Child health, Psychometric validation, Botulinum toxin

## Abstract

**Background:**

The Questionnaire on Pain caused by Spasticity (QPS) is a modular patient- and observer-reported outcome measure of spasticity-related pain (SRP) in children with cerebral palsy (CP). Originally developed for an English-speaking population, we conducted a psychometric validation of a recently developed Chinese language version of the QPS.

**Methods:**

This was a prospective, observational study involving 137 children/adolescents with CP and upper and/or lower limb spasticity and their parents at three sites in China. Six QPS modules were used, three each for upper and lower limb SRP assessment: a patient self-report module; an interviewer-administered module used by site staff based on the cognitive, communicative, and motor abilities of a patient; and a parent/caregiver module administered for all children as an observer-reported outcome to complement the patient-reported outcome. If no assessment by the patient was possible because of age or cognitive impairments, only the parent/caregiver module was completed. Two visits with a 3-week interval provided data to evaluate and establish administrative ease of use, scoring of the QPS (factor analyses, Rasch analyses), reliability (Cronbach’s α, intraclass correlation coefficient), validity (correlations with quality of life [PedsQL™], motor impairment [Gross Motor Function Classification System, Gross Motor Function Measure-66, Manual Ability Classification System], and spasticity [Ashworth Scale, Modified Tardieu Scale]).

**Results:**

For most children, clinic staff reported no difficulties associated with general QPS use or deciding which module to use. Children (and parents) who reported more demanding activities also reported higher levels of associated SRP (or observed SRP behavior). Activity-related SRP items were combined for a total QPS score. Cronbach’s α was low for child self-report, but was acceptable for interviewer-administered and parent reports on SRP. Test–retest reliability was high for all modules. Moderate–strong associations were frequently seen between QPS and quality of life, and were particularly strong in the child self-report group. Relatively weak associations were observed between QPS and motor impairment and spasticity.

**Conclusions:**

This first study was successful in providing initial evidence for the psychometric properties. Clinic staff were able to administer the QPS modules easily, and both children and parents were able to complete the designated QPS appropriately.

**Electronic supplementary material:**

The online version of this article (10.1186/s12955-017-0804-8) contains supplementary material, which is available to authorized users.

## Background

Pain in children and adolescents with cerebral palsy (CP) is common (14%–60%), but poorly understood, infrequently recognized and, therefore, often undertreated [1–5]. The high prevalence of pain in this patient population is associated with poor quality of life (QoL), with QoL scores typically low across all domains of the KIDSCREEN-52 health related QoL questionnaire [6–8]. Due to the range of impairments and disabilities experienced, particularly those related to the musculoskeletal system, pain can arise from multiple sources (e.g. musculoskeletal, chronic constipation, gastroesophageal reflux disease, stomatological diseases, etc.) [3, 8]. One of the major movement disorders in CP is spasticity [9–12], defined as muscular tightness and spasms that can lead to deformities, contractures, and dislocations [3]. Thus, one potential source of pain experienced by this population is spasticity-related pain (SRP). SRP can be continuous or recurrent and the incidence and severity of the pain may be affected by movement and different activities throughout the day (e.g., resting, walking, playing, or exercising) [1, 2, 13, 14].

There are currently no well-validated scales for the assessment of SRP in pediatric or adult populations with CP and only general measures exist [15]. The Questionnaire on Pain caused by Spasticity (QPS) was developed to provide an innovative, standardized, patient-reported outcome (PRO) measure that could be used to describe SRP in a heterogeneous population with CP as described in a prior publication [15]. Furthermore, it was anticipated that such a tool might facilitate the evaluation of potential new therapies for the treatment of SRP in children and adolescents with CP; e.g., botulinum toxin, which has been reported to help with pain in adult spasticity [16–18]. Originally developed for an English-speaking population, several language versions including a Chinese version of the QPS have recently been developed [19].

**Fig. 1:**
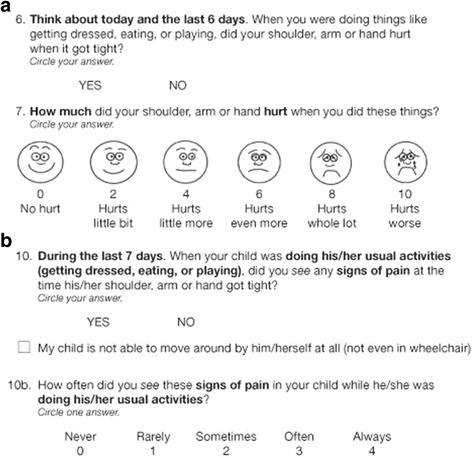
Item examples of the QPS. **a** Item 6 and 7 of the upper extremity child/adolescent module. **b** Item 10 of the upper extremity parent/caregiver module

The QPS was developed following recommended methodology for PROs and Observer-Reported Outcomes (ObsROs) [20–23], including concept elicitation with children and caregivers, item generation with clinician expert input, and refinement of the modules via cognitive interviews in the target population [15, 24–26]. The questionnaire is designed to be used by parents/caregivers and their children/adolescents (2–17 years of age) with CP who experience limb SRP. To accommodate the wide variation in communication skills among patients with CP, three QPS modules were developed, with each available for upper limb (UL) and lower limb (LL) SRP assessments (a total of six modules): a self-report module for children/adolescents (12 items), an interviewer-administered module for children/adolescents (12 items), and an observer-report module for parents/caregivers (13 items). Information gained from the ObsRO for parents and caregivers was designed to complement the information gained from the child/adolescent assessment. The QPS items ask for SRP at rest, during usual daily activities, active mobilization, and a self-defined physically difficult activity (Fig. 1). Whereas children/adolescents report on their experienced SRP severity for these situations (6- point response scale, 0 = ‘no hurt’ - 10 = ‘hurts worst’), parents/caregivers are asked to report observed SRP locations, behaviors and the frequency of SRP (5-point response scale, 0 = ‘never’ to 4 = ‘always’) for the same activity situations used to capture the child/adolescent reports of pain severity (i.e., at rest, during usual daily activities, active mobilization, and a self-defined physically difficult activity).

The objectives of the observational validation study reported here were to establish the first psychometric characteristics of the QPS, specifically regarding scoring, reliability, and validity in a population of Chinese children and adolescents with CP. Furthermore, the study provides supportive information on administration procedures for the questionnaire in this population, and provides an insight into the SRP reported by the children/adolescents and observed by their caregivers.

## Methods

### Study design and population

This was a prospective, observational, one-arm, psychometric validation study of a health outcome measure. The a priori construct validity hypothesis of the study was that patients with greater disability or spasticity would experience a greater level of SRP and a lower QoL.

Children or adolescents with CP and their parents/caregivers were recruited between November 2013 and June 2014 from three hospital sites in China: the Foshan Nanhai Affiliated MCH Hospital of Guangzhou, the Hunan XiangYaBoAi Rehabilitation Hospital, and the Dongguan MCH Hospital. Sites recruited from both their inpatient caseload and their outpatient caseload. The study adhered to the ethical principles for human research and approval was provided by the ethical review board at each participating hospital. Each patient or their legally acceptable representative provided written informed consent to participate in the study.

Male or female patients were included in the study if they were between 2 and 17 years of age with either uni- or bilateral CP, UL and/or LL spasticity, and intermittent SRP in either the ULs or LLs on a weekly basis in one of the following clinical patterns: pes equinus, adducted thigh, flexed knee, flexed elbow, or flexed wrist. Patients were also required to have an Ashworth Scale (AS) score of ≥1 in at least one of the pre-defined clinical patterns and were receiving continuous anti-spastic treatment and/or medication. Key exclusion criteria included fixed contractures, predominant forms of muscle hypertonia other than spasticity, constant SRP over very long time periods, LL and/or UL surgery within the past 12 months, and an indication for orthopedic surgery within the next 2 months.

The study planned to recruit 135 child/adolescent together with one parent/caregiver. To enable all key ages to be covered by the QPS modules, three specific groups of children/adolescent–parent pairs were recruited: 1) 120 patients with LL and/or UL SRP who could self-complete the QPS or were able to use the interviewer-administered module; 2) 45 of these 120 patients with UL SRP (this subset of group 1 was included to account for the lower prevalence of UL SRP vs LL SRP among patients with CP); and 3) 15 patients with LL or UL SRP who were incapable of using the QPS (i.e., the parent/caregiver QPS module was used because either the patient was too young or he/she had disabilities that precluded him/her from participating in the assessment).The sample size for these 135 child/adolescents with their parents was based on assumptions including representation of population heterogeneity, number of items and power for convergent validity testing (e.g. *n* = 75 for 95% power of showing a moderate correlation of 0.4).

### Assessments

The study (No.: MRZ99901_0027_4) comprised two clinic visits, a screening/baseline visit (V1/V2) and an end-of-study visit (V3) scheduled for 21 (±3) days later. The QPS was administered at the baseline (V2) and end-of-study visits (V3) at site. Based on the pre-screening of the cognitive, communicative, and motor abilities of a patient, the QPS was self-administered by the patient, or the interviewer-administered module was used by site staff. If no assessment by the patient was possible, due to age or cognitive impairments, only the parent/caregiver module was completed. The parent/caregiver module of the QPS (ObsRO) was completed for each child by the same parent/caregiver throughout the study. The parent/caregiver had regular contact with his/her child/adolescent so that he/she could report reliably on the observed SRP behaviors. Alongside the QPS assessment, the clinic staff responsible for the QPS assessment procedure were asked to complete a questionnaire documenting ease of use of the QPS (e.g. difficulties in selection of modules for children) and any administration issues (e.g. encountered problems and positive experiences).

In addition to the QPS, a number of standard clinical evaluations for the study population and for spasticity were performed during the study. As standard classifications in the CP population, the Gross Motor Classification System – Expanded and Revised [GMFCS] and the Manual Ability Classification System [MACS]) were used [27–29]. The GMFCS is a classification of general motor independence with five classification levels (Level I: Walks without limitations – Level V: Transported in a manual wheelchair). The MACS is a classification for CP children with five levels on the self-initiated abilities to handle objects and their need for assistance or adaptation to perform manual activities (Level I: Handles objects easily and successfully – Level V: Does not handle objects and severely limited ability to perform even simple actions). For evaluation of motor function, the Gross Motor Function Measure-66 [GMFM]) was used, which utilizes 66 functional tests that span the spectrum from activities in lying and rolling up to walking, running and jumping skills [30]. Cognitive ability by means of cognitive age was based on the standard tools used by sites (e.g. Gesell Developmental Schedule, Sign-Significant Relations Rehabilitation Rating). The level of spasticity in spastic joints in this study was determined with the AS and Modified Tardieu Scale [MTS]) [31–33]. Spasticity with the AS is assessed during passive range of motion of a joint on a 5-point response scale (‘0 = ‘No increase in tone’ - ‘4 = ‘Limb rigid in flexion or extension’). For the MTS, the spasticity angle was documented, which is the difference between the passive full range of motion angle with slow assessment velocity minus the fast stretch speed angle of a spastic joint. The Pediatric Quality of Life Inventory™ (PedsQL™) was used to capture information on patient QoL [34, 35].There are four sub-scales that refer to physical functioning (8 items), emotional functioning (5 items), social functioning (5 items), and school functioning (5 items). The association of the QPS to overall Quality of Life was of main interest, and hence the total score of the PedsQL™ was the focus for the analyses. Patient demographic data and CP history were also recorded.

For all assessment tools, the official Chinese language versions were used during the study. The Chinese version of the QPS was established using a standard linguistic validation process for developing new language versions for data collection measures [19, 36]. The process included forward and backward translation steps, and cognitive interviews with Chinese children with CP and SRP and their parents/caregivers. Finally, an international harmonization step was used for quality control and finalization of the translations.

### Statistical analyses

The demographic characteristics of the children/adolescents and their parents were summarized using descriptive statistics, controlling for the actual QPS modules used (LL and/or UL) and whether the QPS was self- or interviewer-administered.

To evaluate the individual QPS items, responses were evaluated by means of standard item-reduction statistics (e.g., ceiling/floor effects, missing data, item-to-item correlation, corrected item-total correlation (0.40 threshold) [37]. Factor analyses (principal component analysis with varimax rotation using Kaiser normalization) were conducted to assess scale dimensionality with factor loadings of at least 0.40 as a cut-off [38]. Rasch Measurement Theory (RMT, one-parameter logistic model, RUMM2030 software) [39] models were utilized to review item functioning of tested constructs. Due to small sample sizes, RMT models were only run for the sample of children with LL SRP using combined results from self-reported and interviewer modules. Based on these results, the final QPS items were selected and a scoring algorithm was created. The following validation analyses of the QPS were then performed based on the final scoring approach.

Internal consistency of the QPS was evaluated by Cronbach’s α analysis using 0.70 as the desired threshold [40]. Test–retest reliability between both visits (V2 and V3) using total score and single items, was demonstrated by intraclass-correlation coefficient (ICC 2,1) estimates, using 0.70 as the desired threshold [41].

Convergent/discriminant validity was investigated by Pearson correlations of QPS variables with other measures of interest, such as GMFCS, AS and MTS scores, GMFM, and PedsQL™ using associations of at least 0.30 as desired level of association. Construct validity of the QPS was tested by comparison of reported SRP levels in relation to activities and differences in important demographic groups in the study population such as the GMFCS or MACS categories. Specifically, higher levels of SRP to QoL based on the available studies were expected [6–8]. Also higher SRP levels were hypothesized to be associated with higher degrees of spasticity (AS, MTS) and disability (GMFM, GMFCS, MACS), but prior studies have indicated not always clear associations in this regard [4, 8, 14]. In addition, ordinal logistic regression analysis models were run with a set of potential predictors for identification of important SRP-related factors. All analyses, with the exception of RMT, were conducted using SPSS [42].

## Results

### Population

A total of 137 children/adolescents with CP and their parents/caregivers were enrolled and contributed to this study, from which all 137 completed it. None of the children had missing QPS data and only a very small amount of QPS data were missing for the parents/caregivers (maximum 3 cases, 2.4%). Some of the clinical assessments had small amounts of missing data (i.e., AS 21 cases, 15%; MTS 20 cases, 14%).

**Fig. 2:**
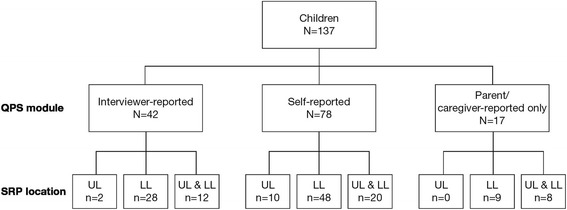
Number of children recruited and analysis sets based on SRP location and QPS module used. LL, lower limb; UL, upper limb; SRP, spasticity-related pain

Most children presented with bilateral LL spasticity (117/137, 85.4%). Although 52 children presented with UL spasticity overall, UL spasticity occurred most often together with LL spasticity and very few (10/137, 7.3%) presented with UL spasticity alone (Fig. 2).

The study population covered the full age spectrum (range 2–17 years) with a mean (standard deviation, SD) age of 6.6 (3.2) years. Most children were 4–8 years of age; 68.6% were male and 93.4% were Chinese (Table 1). Children who could self-administer the QPS had a mean age of 7.9 (3.1) years, those who completed the interviewer-administered QPS had a mean age of 5.3 (2.3) years and those who couldn’t use the QPS had a mean age of 3.8 (1.5) years.

**Table 1:** Demographic characteristics of children and parents at baseline visit (V2)

		Children, *N* = 137	Parents, *N* = 137
Age, years	Mean (SD, range)	6.6 (3.2, 2.0–17.5)	39.6 (11.0, 24.9–69.1)
Gender, *n* (%)	Male	94 (68.6)	23 (16.8)
	Female	43 (31.4)	114 (83.2)
Education Category, *n* (%)	Elementary school	136 (99.3)	32 (23.4)
	High school	1 (0.7)	75 (54.7)
	University	–	30 (21.9)
Ethnic group, *n* (%)	Chinese	128 (93.4)	129 (94.2)
	Non-Chinese^a^	9 (6.6)	8 (5.8)
Age at CP diagnosis, years	Mean (SD, range)	1.8 (1.7, 0.1–10)	
Age at spasticity diagnosis, years	Mean (SD, range)	1.9 (1.6, 0.1–10)	
Main Cause of CP, *n* (%)^b^	Premature delivery	70 (51.1)	
	Lacking oxygen	46 (33.6)	
	Low birth weight	13 (9.5)	
	Brain conditions	9 (6.6)	
	Reason unclear	12 (8.8)	
	Other causes	25 (18.2)	
GMFCS, *n* (%)	Level I – Walks without limitations	27 (19.7)	
	Level II – Walks with limitations	46 (33.6)	
	Level III –Walks using a hand-held mobility device	29 (21.2)	
	Level IV –Self-mobility with limitations; may use powered mobility	13 (9.5)	
	Level V – Transported in a manual wheelchair	22 (16.1)	
MACS, *n* (%)	Level I – Handles objects easily and successfully	40 (29.2)	
	Level II – Handles most objects but with somewhat reduced quality and/or speed of achievement	49 (35.8)	
	Level III – Handles objects with difficulty; needs help to prepare and/or modify activities	30 (21.9)	
	Level IV – Handles a limited selection of easily managed objects in adapted situations	10 (7.3)	
	Level V – Does not handle objects and has severely limited ability to perform even simple actions.	4 (2.9)	
	Missing	4 (2.9)	

*CP* cerebral palsy, *GMFCS* Gross Motor Function Classification System - Expanded and Revised, *MACS* Manual Ability Classification System, *SD* standard deviation

^a^Refers to ethnicity/country of birth; ^b^Multiple answers possible

The mean (SD) age of the parents/caregivers was 39.6 (11.0) years (range 24.9–69.1 years) and the majority (83.2%) were female. Most parents were described as not employed outside the home (50.4%) or homemakers (19.0%). The greatest proportion of children (33.6%) were Level II on the GMFCS and 35.8% were Level II on the MACS (Table 1). Overall, the study sample reflected a heterogeneous one, as indicated by the GMFCS classification.

### QPS: Key problems and ease of use

To provide information on the ease of use of the QPS in daily practice and identify any administration issues, clinic staff were asked to complete a questionnaire regarding any difficulties. No difficulties were reported in deciding which module of the QPS to use for most (117/137, 85%) children and difficulties related to uncertain cognitive abilities were reported for only a few children (15/137, 10.9%). Furthermore, clinic staff noted no general administration difficulties for most of the children (80/137, 58.4%), although, some difficulties in assigning pain and stiffness using QPS items 1 and 2 were reported (45/137, 32.8% of children), and when defining SRP in general (11/137, 8.0% of children). Based on this experience, for approximately one third of children (49/137, 35.8%), the clinic staff mainly focused on cognitive ability and ability to communicate/express the location of their SRP when deciding on the appropriate module to use.

### QPS item analysis

The mean QPS score patterns of the items that contribute to the QPS are shown in Table 2. Similar mean score patterns were noted for all the different child/adolescent QPS modules with relatively high SRP levels associated with exercise (physical therapy or stretching exercises) followed by the general item of SRP when tight. Low levels of SRP were expressed while at rest (sitting, watching TV, or trying to sleep). Similar to the child results, the greatest amount of pain observed by the parent occurred when the child was exercising. Almost no pain was noted by parents when the children were at rest.

**Table 2:** Important single item results of QPS modules at baseline visit (V2)

QPS module, item number and description	Mean (SD)	Range	Floor *N* (%)	Ceiling *N* (%)	Missing *N* (%)	Item-Total Correlation ^c^	Cronbach’s α ^d^
Child self LL n = 68 ^a^							
3. How much did your <*limb* > hurt when tight	4.4 (2.7)	0–10	5 (7.4%)	5 (7.4%)	0 (0.0%)	–	–
5. How much did your <*limb* > hurt when sitting	0.2 (0.7)	0–4	61 (89.7%)	0 (0.0%)	0 (0.0%)	.322**	0.619
7. How much did your <*limb* > hurt when moving	1.0 (1.7)	0–6	49 (72.1%)	0 (0.0%)	0 (0.0%)	.751**	0.385
9. How much did your <*limb* > hurt during exercise	5.8 (2.8)	0–10	2 (2.9%)	10 (14.7%)	0 (0.0%)	.761**	0.656
12. How much did your <*limb* > hurt hard	1.2 (1.8)	0–6	43 (63.2%)	0 (0.0%)	0 (0.0%)	.788**	0.337
Child self UL n = 30 ^a^							
3. How much did your <*limb* > hurt when tight	4.4 (2.7)	0–10	2 (6.7%)	2 (6.7%)	0 (0.0%)	–	–
5. How much did your <*limb* > hurt when sitting	0.2 (0.7)	0–4	29 (96.7%)	0 (0.0%)	0 (0.0%)	.392*	0.227
7. How much did your <*limb* > hurt when moving	1.0 (1.7)	0–4	25 (83.3%)	0 (0.0%)	0 (0.0%)	.487**	0.168
9. How much did your <*limb* > hurt during exercise	5.8 (2.8)	0–10	2 (6.7%)	2 (6.7%)	0 (0.0%)	.653**	0.454
12. How much did your <*limb* > hurt hard	1.2 (1.8)	0–10	20 (66.7%)	1 (3.3%)	0 (0.0%)	.745**	0.052
Child interviewer LL n = 40 ^a^							
3. How much did your <*limb* > hurt when tight	3.6 (2.5)	0–8	7 (17.5%)	0 (0.0%)	0 (0.0%)	–	–
5. How much did your <*limb* > hurt when sitting	2.0 (2.5)	0–8	22 (55.0%)	0 (0.0%)	0 (0.0%)	.817**	0.701
7. How much did your <*limb* > hurt when moving	3.0 (2.2)	0–8	8 (20.0%)	0 (0.0%)	0 (0.0%)	.734**	0.755
9. How much did your <*limb* > hurt during exercise	6.6 (2.5)	0–10	1 (2.5%)	4 (10.0%)	0 (0.0%)	.789**	0.734
12. How much did your <*limb* > hurt hard	3.3 (1.9)	0–8	4 (10.0%)	0 (0.0%)	0 (0.0%)	.777**	0.715
Child interviewer UL *n* = 14 ^a^							
3. How much did your <*limb* > hurt when tight	3.6 (2.5)	0–8	2 (14.3%)	0 (0.0%)	0 (0.0%)	–	–
5. How much did your <*limb* > hurt when sitting	2.0 (2.5)	0–6	10 (71.4%)	0 (0.0%)	0 (0.0%)	.886**	0.899
7. How much did your <*limb* > hurt when moving	3.0 (2.2)	0–6	7 (50.0%)	0 (0.0%)	0 (0.0%)	.867**	0.886
9. How much did your <*limb* > hurt during exercise	6.6 (2.5)	2–8	0 (0.0%)	0 (0.0%)	0 (0.0%)	.913**	0.880
12. How much did your <*limb* > hurt hard	3.3 (1.9)	0–6	2 (14.3%)	0 (0.0%)	0 (0.0%)	.908**	0.860
Parent LL, *n* = 125 ^b^							
2. Hours/day spent in direct contact	17.9 (8.2)	1–24	1 (0.8%)	76 (60.8%)	1 (0.8%)	–	–
8. Often seen signs of pain when tight	1.7 (1.0)	0–4	20 (16.0%)	0 (0.0%)	1 (0.8%)	–	–
9b. Often seen signs of pain at rest	0.4 (0.6)	0–3	90 (72.0%)	0 (0.0%)	0 (0.0%)	.679**	0.699
10b. Often seen signs of pain activities	0.9 (0.9)	0–3	51 (40.8%)	0 (0.0%)	2 (1.6%)	.727**	0.693
11b. Often seen signs of pain exercise	2.7 (1.0)	0–4	3 (2.4%)	0 (0.0%)	0 (0.0%)	.801**	0.639
13b. Often seen signs of pain hard thing	1.3 (1.1)	0–4	36 (28.8%)	0 (0.0%)	0 (0.0%)	.800**	0.674
Parent UL, n = 52 ^b^							
2. Hours/day spent in direct contact	19.3 (7.8)	2–24	0 (0.0%)	37 (71.2%)	0 (0.0%)	–	–
8. Often seen signs of pain when tight	1.6 (1.0)	0–4	8 (15.4%)	0 (0.0%)	0 (0.0%)	–	–
9b. Often seen signs of pain at rest	0.3 (0.5)	0–2	41 (78.8%)	0 (0.0%)	0 (0.0%)	.531**	0.709
10b. Often seen signs of pain activities	1 (0.9)	0–3	20 (38.5%)	0 (0.0%)	1 (1.9%)	.738**	0.627
11b. Often seen signs of pain exercise	2.4 (0.9)	1–4	0 (0.0%)	0 (0.0%)	0 (0.0%)	.772**	0.585
13b. Often seen signs of pain hard thing	1.3 (1.2)	0–4	18 (34.6%)	0 (0.0%)	0 (0.0%)	.842**	0.573

QPS score items are items 5, 7, 9 and 12 for child modules and items 9b, 10b, 11b, and 13b for parent modules

QPS modules: Self, self-administered; Interviewer, interviewer-administered

*LL* lower limb, *UL* upper limb, *SD* standard deviation

^a^Child/adolescent QPS score range: 0 = ‘no hurt’ to 10 = ‘hurts worst’

^b^Parent QPS score ranges: Item 2, 0–24 h; Items 8–13b, 0 = ‘never’ to 4 = ‘always’

^c^corrected Pearson correlations for QPS score; **, significance at 0.01 level

^d^if item is missing for QPS score

The low SRP levels in the study population for the ‘SRP at rest’ items and in some instances for the ‘SRP doing usual activities’ items, resulted in floor effects for these items. This was also reflected in the item-to-item and corrected item-total correlations (Table 2 and Table 3). Conversely, the pain levels supported the hypothesis that easier activities should be related to lower SRP levels than more difficult activities. Since the qualitative work for the QPS creation of these items was shown to be of relevance for some children and their parents, and the SRP levels followed the hypothesized pattern, the items were included in the subsequent scoring analyses. No ceiling effects were found for any of the QPS items and the entire spectrum of the scale was seen, with high SRP levels being reported in some patients.

**Table 3:** QPS item-to-item and item-to-total correlations (Pearson correlations)

QPS module	Item-to-item correlation range		Item-to-total correlations
	General pain item	Other four activity items	
Child self LL (*n* = 68)	−0.037 to 0.611	0.099 to 0.705	0.322 to 0.788
Child self UL (*n* = 30)	0.003 to 0.698	−0.022 to 0.702	0.392 to 0.745
Child interviewer LL (*n* = 40)	0.178 to 0.490	0.316 to 0.549	0.734 to 0.817
Child interviewer UL (*n* = 14)	0.387 to 0.754	0.676 to 0.859	0.867 to 0.913
Parent LL (*n* = 125)	0.263 to 0.464	0.359 to 0.554	0.679 to 0.801
Parent UL (*n* = 52)	0.304 to 0.526	0.179 to 0.640	0.531 to 0.842

QPS items reported are items 3, 5, 7, 9 and 12 for child modules and items 8, 9b, 10b, 11b, and 13b for parent modules

QPS modules: Self, self-administered; Interviewer, interviewer-administered

*LL* lower limb, *UL* upper limb

### QPS measurement model and scoring

Three scoring algorithms were explored with the data to establish a score for the QPS and included testing to include all QPS items and different combinations of SRP items. In the final scoring system, based on optimal results (IRT and factor analyses), the item on general SRP was removed from the overall score, keeping it as an external overall criterion. The four remaining activity-related SRP items (at rest, usual activities, with exercise and when trying to do a specified hard task) were included to calculate the QPS score. This decision was supported by IRT and factor analyses, in which the general item displayed significant misfit, whereas the other items performed well (50–70% of variance). The other items of the QPS contribute additional information relating to the established overall score. In the children modules they are a useful check of response consistency. In the parent modules, the other items provide further information on the localization of SRP and definition of SRP behaviors [15].

Within the UL modules (self-administered child/adolescent and parent), there were still two 2-dimensional results and not the overall anticipated uni-dimensional score. Here, the exercise item contributed to a second factor, which fitted the assumption that exercise is always reported with a high level of pain. Because all the other factor analyses yielded a uni-dimensional structure it was decided to adopt the single-score approach (the pain level for exercise activity was high here as well), and to revisit these issues when future QPS data become available for analyses.

**Fig. 3:**
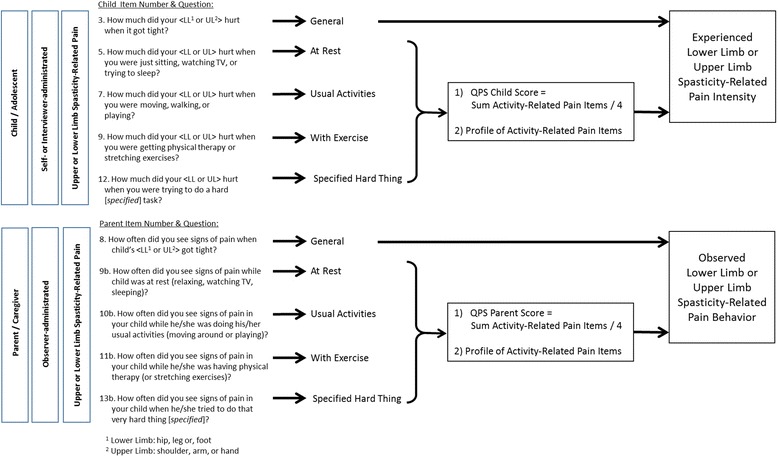
Conceptual framework and scoring of the QPS

An overview of the final scoring approach and the conceptual framework of the QPS is presented in Fig. 3. Although not contributing to the score, the general item was reported together with the single activity-related items, to allow reporting of the overall SRP profile of a child with CP and as a general external reference.

Furthermore, separate reporting of the items and QPS score was recommended for the six modules to account for the different CP etiologies and age groups (i.e., a higher proportion of self-administered modules than interviewer-administered modules were expected to be completed in older children).

### QPS reliability

Using the newly defined QPS score, internal consistency (Cronbach’s α) and test–retest reliability (ICC 2,1) were tested (Table 4). Although not presented in detail in this paper, clinical measures such as the AS remained stable between visits, thus supporting an appropriate setting for testing reliability. While Cronbach’s α was lower than expected for the child self-report group, the coefficients for the child interviewer-administered group were adequate (>0.70, 0.78 for LL and 0.91 for UL) [43]. Parent modules also showed acceptable consistency (0.74 for LL and 0.70 for UL). Test–retest reliability was 0.92 to 0.95 for the QPS score, demonstrating ‘almost perfect’ (>0.81) results [43]. Also for single items, test–retest reliability results were ‘substantial’ (0.61 to 0.80) or ‘almost perfect’ between the 3-week interval of the two study visits (V2 and V3).

**Table 4:** Cronbach’s α at baseline visit (V2) and test–retest reliability of QPS score V2–V3

QPS module	Mean (SD), V2	Internal consistency, V2 (Cronbach’s α)	Test–retest reliability, V2–V3 (ICC 2,1)	
	QPS score	QPS score	QPS score	Single items^a^
Child self LL (*n* = 68)	2.0 (1.3)	0.579	0.942	0.89–0.91
Child self UL (*n* = 30)	1.6 (1.2)	0.281	0.925	0.90–0.96
Child interviewer LL (*n* = 40)	3.7 (1.8)	0.780	0.940	0.82–0.93
Child interviewer UL (*n* = 14)	2.6 (1.9)	0.906	0.972	0.86–0.99
Parent LL (*n* = 122–125)	1.3 (0.7)	0.738	0.959	0.90–0.94
Parent UL (*n* = 51–52)	1.2 (0.7)	0.699	0.919	0.78–0.91

QPS modules: Self, self-administered; Interviewer, interviewer-administered

*LL* lower limb, *UL* upper limb

^a^QPS items reported are items 5, 7, 9 and 12 for child modules and items 9b, 10b, 11b, and 13b for parent modules

### QPS validity

With regard to construct validity of the QPS score items, as stated earlier, scores were consistently higher for more difficult activities, with the ‘at rest’ item related to the lowest scores and the ‘exercise’ item to the highest scores (Table 2). For further investigation of convergent/construct validity with other concepts of interest, correlations with the QPS score were performed and QPS scores across groups were explored. According to the 5-point AS rating conducted by the clinicians, SRP was widely reported across the spasticity grades in the main muscle group patterns by children and parents. Thus, associations in the hypothesized direction were not found. In contrast, there was a trend towards some moderate-to-high associations in the hypothesized direction between the QPS score and MTS spasticity angle (Tables 5).

**Table 5:** QPS Score correlation to AS and MTS at baseline visit (V2)

QPS module	AS, mean of main muscle group^a^		MTS^b^			
Expected association	Higher AS score relates to greater spasticity impairment and higher QPS score		Larger MTS spasticity angle relates to greater spasticity impairment and higher QPS score			
	LL	UL	Adducted thigh	Pes equinus	Flexed wrist	Flexed elbow
Child self LL (n = 68)	−0.068	0.052	**0.310**	0.198	0.319	0.136
Child self UL (n = 30)	−0.468	−0.212	**0.455**	0.304	0.234	0.042
Child interviewer LL (n = 40)	−0.244	−0.245	**0.430**	**0.448**	0.261	−0.224
Child interviewer UL (*n* = 14)	−0.266	−0.486	0.111	**0.771**	−0.418	−0.555
Parent LL (n = 125)	**−0.242**	**−0.476**	**0.335**	0.112	0.227	−0.036
Parent UL (n = 52)	**−0.574**	**−0.353**	**0.435**	**0.582**	0.153	−0.190

QPS Score correlation to the AS for main muscle group angles and MTS for two joints of the upper and lower limb (Pearson correlation coefficients at baseline visit [V2])

Significant associations are shown in bold (*p <* 0.05)

QPS modules: Self, self-administered; Interviewer, Interviewer-administered

*AS* Ashworth Scale, *LL* lower limb, *MTS* Modified Tardieu Scale, *UL* upper limb

^a^AS scores were combined for each child based on main SRP patterns, as indicated by the investigator. AS score is based on passive range of motion assessment of a joint: 0 = ‘No increase in tone’; 1 = ‘Slight increase in tone giving a ‘catch’ when the limb was moved in flexion or extension’; 2 = ‘More marked increase in tone, but limb easily flexed’; 3 = ‘Considerable increase in tone – passive movements difficult’; 4 = ‘Limb rigid in flexion or extension’

^b^The MTS spasticity angle is the difference between the full range of motion with slow assessment velocity of a joint minus the fast stretch speed angle. MTS data for the left body side is presented for demonstration

For the association between QPS and motor function, as measured by the GMFCS and GMFM-66, some conflicting (child LL self-report group), but also clear relations were seen (child interviewer group, Table 6 and Additional file 1: Figure S1). This was not the case for the parent questionnaire modules and for the association to MACS, which fitted the general notion that SRP was reported across the disability spectrums (Additional file 1: Fig. S1). Contrary to these findings, clear associations were found with QoL, as measured by moderate-to-high associations with the PedsQL™ (Table 6, Additional file 2: Figure S2), which reached significance in the QPS LL category (*p <* 0.01). Although it should be noted that a smaller proportion of those in the child interviewer groups completed the PedsQL™, the overall pattern showed that there seemed to be a close association between reported levels of SRP and general QoL.

**Table 6:** QPS score correlation to GMFM-66, GMFCS, MACS, PedsQL™ (Pearson correlation coefficients at baseline visit [V2])

QPS module	GMFM-66	GMFCS	MACS	PedsQL™ total score^a^
Expected association	Higher GMFM-66 score [0–100] relates to better motor function and lower QPS score	Higher GMFCS/MACS levels [I–V] relate to higher disability and higher QPS score		Higher PedsQL score [0–100] relates to better QoL and lower QPS score
Child self LL	0.258* (n = 68)	−0.242*(*n* = 68)	−0.067 (n = 68)	**−0.325** ^**^ (*n* = 67)
Child self UL	0.185 (n = 30)	−0.288 (*n* = 30)	0.299 (n = 30)	−0.136 (n = 30)
Child interviewer LL	−0.378* (*n* = 40)	0.503** (n = 40)	0.171 (n = 40)	**−0.685** ^**^ (*n* = 21)
Child interviewer UL	−0.485 (*n* = 14)	0.644*(n = 14)	0.150 (n = 14)	−0.861 (n = 4)
Parent LL	−0.052 (*n* = 125)	0.021 (n = 125)	0.017 (n = 125)	**−0.441** ^***^ (*n* = 124)
Parent UL	−0.145 (*n* = 52)	0.023 (n = 52)	0.029 (n = 52)	**−0.375** ^**^ (*n* = 51)

Significant associations are shown in bold, *p < 0.05; **p < 0.01; ***p < 0.001

QPS modules: Self, self-administered; Interviewer, interviewer-administered

*GMFCS* Gross Motor Function Classification System - Expanded and Revised, *GMFM-66* Gross Motor Function Measure-66, *LL* lower limb, *MACS* Manual Ability Classification System, *PedsQL™* Pediatric Quality of Life Inventory, *SD* standard deviation, *UL* upper limb

^a^Correlations of QPS child modules vs PedsQL™ child scores and QPS parent modules versus PedsQL™ parent score

To explore QPS validity further, an exploratory ordinary least squares (OLS) regression model was run on the QPS baseline scores with the following independent variables being included in the model: age, gender, GMFCS, MACS, health state of child (self-report and parent-report), use of assistive devices (yes/no), and therapy (yes/no) [Additional file 3: Table S1]. For ease of analysis and sample size, the self-report and interviewer-administered QPS modules were combined. Most models showed reasonably high variance. Using the child LL model, age, GMFCS, use of assistive devices and therapy had a significant effect on QPS scores, while in the parent LL model GMFCS, overall health of the child, use of assistive devices and therapy had a significant effect on QPS scores (Additional file 3: Table S1). The sample sizes were too small to run the multiple regressions for UL in both the child and parent samples.

## Discussion

The objective of this initial QPS validation study was to establish the scoring and evaluate the psychometric characteristics of this questionnaire, specifically relating to reliability and validity. The recruited study population of 137 children/adolescents and their parents covered the complete age range from 2 to 17 years, the major proportion of the patients comprising younger children. The complete CP motor disability spectrum, according to the GMFCS, was well represented. Most of the children had bilateral spasticity, which is commonly reported, while few had UL spasticity alone [12]. This distribution mirrors the typical rehabilitation setting of children and is a more interesting research population compared with a population that is older, particularly when wishing to test how well the QPS can be utilized in younger children. Thus, the overall study population sample was regarded as being a typical and suitable for QPS testing, specifically for the larger LL spasticity group.

Results of the ease-of-use questionnaire provided highly positive feedback from clinic staff on the administration of the QPS. The majority of reported difficulties related to the age of the children and their ability to understand the aim of the QPS and to self-report. This observation suggests that screening a child’s capabilities is warranted before the first QPS administration. In case of any doubt between self- or interviewer module after the screening process, the interviewer-reported module should be the version of choice. Responses for SRP items followed the expected pattern and confirmed previous results that more demanding activities were related to higher levels of pain [2, 4, 13, 14, 44]. The general pain item did not represent the reported SRP maximum or a mean value of the four activity-related SRP items. This observation suggests that it is more meaningful to enquire about specific activities and situations that matter to the patient, otherwise, information on the true severity and diversity of the pain is lost.

While the responses obtained from the interviewer and parent modules increased with more demanding activities, the distribution for some items relating to easier activities in the self-reported modules were more bi-directional, i.e., either no self-reported SRP or then a higher score was reported. This resulted in floor effects for ‘no’ or ‘low-demand’ activities, and the exercise item served as the prominent SRP-reported item besides the general item.

Together with the less well-represented UL spasticity group, the prominence of the exercise item provided the only other issue in terms of achieving uni-dimensionality for the final chosen QPS scoring algorithm and explaining the results of the item-to-total analyses. However, the final chosen QPS scoring algorithm and conceptual framework were well supported in the majority of the modules and were the most homogenous of all the explored solutions, so can be considered to be robust and suitable for future research and application.

Test–retest reliability between the two study visits separated by a 3-week interval showed excellent properties, with ICC values exceeding 0.9 for all QPS modules [43]. Cronbach’s α analyses for consistency of the score showed acceptable ranges for most modules, but not for the self-report modules in which the results were lower than expected. This result was not completely unexpected, because reliability of response is generally lower for child PROs [45–47]. Consistent to the feedback of clinical staff and parents discussed prior, this result highlight that screening of a child’s capabilities is extremely important and in case of any doubt the interviewer-reported module should be chosen.

Validity was explored by relating QPS scores to clinical assessments of spasticity, motor function, and QoL. In general, the construct of the QPS was well supported by the fact that more demanding activities corresponded to higher levels of reported SRP. Associations were low-to-moderate for the AS; however, some stronger associations were noted with the MTS. It was hypothesized that SRP could be linked to these spasticity measures, but several factors may explain why this was not the case. The AS has only a limited set of response options, and data were similar due to the fact that only children with spasticity were included in the study. MTS adds linearity to the spasticity measurement [48], which may be one reason why stronger associations were evident between QPS score and MTS. Additionally, these clinical measures rely on passive joint- and muscle-tone evaluation, which may be differentiated from experienced spasticity [33, 49]. In line with this, other recent research indicates that change in muscle tone and pain are not related [16].

Increasing QPS SRP levels were associated with a decrease in motor function as assessed by the GMFCS, especially for LL-reported SRP. In addition, the OLS model on QPS baseline scores showed that age, GMFCS, use of assistive devices and therapy had a significant effect on QPS scores for the child LL group. GMFCS, overall health of the child, use of assistive devices and therapy were also predictive in the parent LL group. Interestingly, QPS scores for the child self-report LL group and child interviewer-administered LL group were also significantly associated with gross motor function as measured by the GMFM-66 score. Other studies have reported a mixed picture on these associations, with some reporting that pain in children with CP is widely distributed across motor impairment levels [4, 14], and others suggesting a dependency [8, 50]. Our results suggested that for specific sub-groups of the study population such an association exists, with higher motor limitation being associated with higher pain levels.

Clear associations were expected for the association between the QPS and QoL, as pain has significant effects on QoL in children/adolescents with CP [6–8, 14]. Moderate-to-strong associations between child and parent SRP and QoL (PedsQL™) were frequent and specifically dominant within the larger sample size groups in the LL category. Based on the general importance of pain for QoL, these results support the convergent validity of the QPS [1, 5, 13].

Study limitations included a highly diverse target population with respect to spasticity patterns, motor disability, cognition, and age. Thus, small sample sizes in this study particularly for patient-sub groups such as patients with UL spasticity are a study limitation, although the distribution of UL spasticity to LL spasticity does reflect the typical etiology of the CP population. The fact that the study only included patients with SRP may have introduced bias towards SRP as the concept of interest, but this was necessary to conduct our research. With testing of the QPS being limited to Chinese children, cultural differences may have influenced outcomes for the different SRP activity items [51], which may be of interest for future research. Finally, there is a need to demonstrate the sensitivity, i.e., responsiveness to change of the QPS (e.g. after botulinum toxin injection) to the effects of spasticity treatment in an appropriately designed interventional study.

## Conclusion

This first study conducted, in China, successfully provided initial evidence for the psychometric properties of the QPS. QPS scores were highly reliable and reproducible. Good convergent validity of the QPS was demonstrated for QoL and motor impairment. No clear association was found for clinically rated spasticity. Although further data are required to bolster our findings, such as sensitivity analyses and exploration of the differences between modules found in the study population, the initial reliability and validation results are encouraging and support the QPS as a reliable and valid measure of SRP in patients with CP. The QPS can be obtained by emailing the corresponding author.

## Additional files

Additional file 1: Figure S1. Mean (SD) QPS scores of the six modules in relation to GMFCS levels. GMFCS, Gross Motor Function Classification System; SD, standard deviation. (JPEG 54 kb)

Additional file 2: Figure S2. QPS score relationship to PedsQL™, PedsQL™, Pediatric Quality of Life Inventory. (JPEG 73 kb)

Additional file 3: Table S1. Ordinal logistic regression analysis model of variables influencing QPS scores at baseline visit (V2). (DOCX 13 kb)

## Abbreviations

AS: Ashworth Scale
CP: cerebral palsy
GMFCS: Gross Motor Function Classification System – Expanded and Revised
GMFM: Gross Motor Function Measure-66
IRT: Item Response Theory
LL: lower limb
MACS: Manual Ability Classification System
MTS: Modified Tardieu Scale
ObsRO: observer-reported outcome
OLS: ordinary least squares
PedsQL™: Pediatric Quality of Life Inventory™
PRO: patient-reported outcome
QoL: quality of life
QPS: Questionnaire on Pain caused by Spasticity
SRP: spasticity-related pain
UL: upper limb
