# Supplementary figures and images for: Initial psychometric validation of the questionnaire on pain caused by spasticity (QPS)
Source: Health Qual Life Outcomes. 2017 Nov 28;15:229. doi: 10.1186/s12955-017-0804-8 (PMC5704623; doi:10.1186/s12955-017-0804-8)

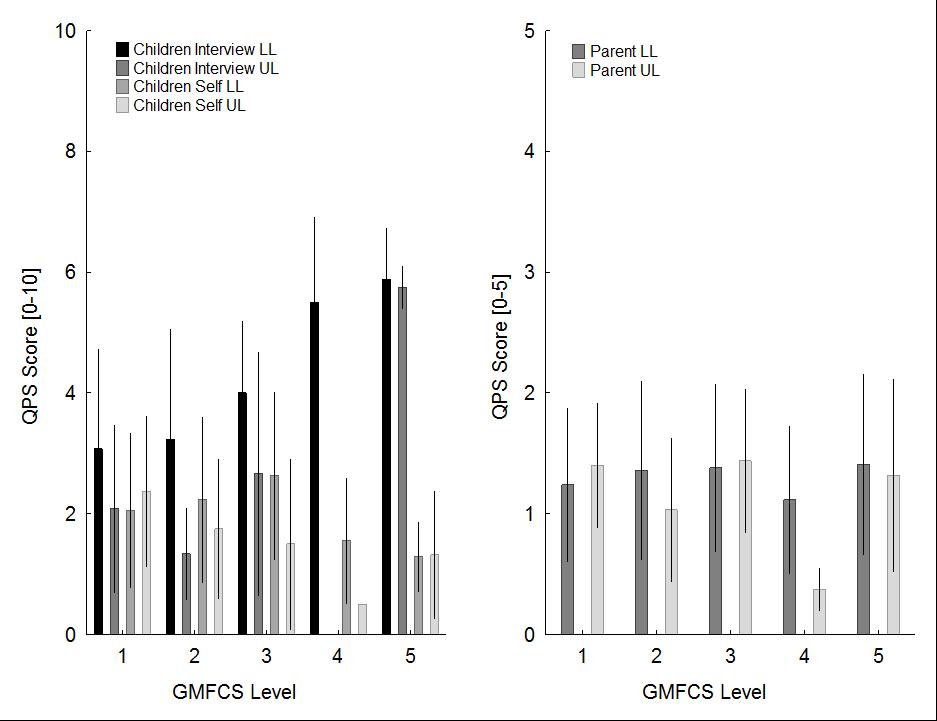

Supplement: Supplementary file 1 — Mean (SD) QPS scores of the six modules in relation to GMFCS levels. GMFCS, Gross Motor Function Classification System; SD, standard deviation. (JPEG 54 kb) [file 12955_2017_804_MOESM1_ESM.jpg]

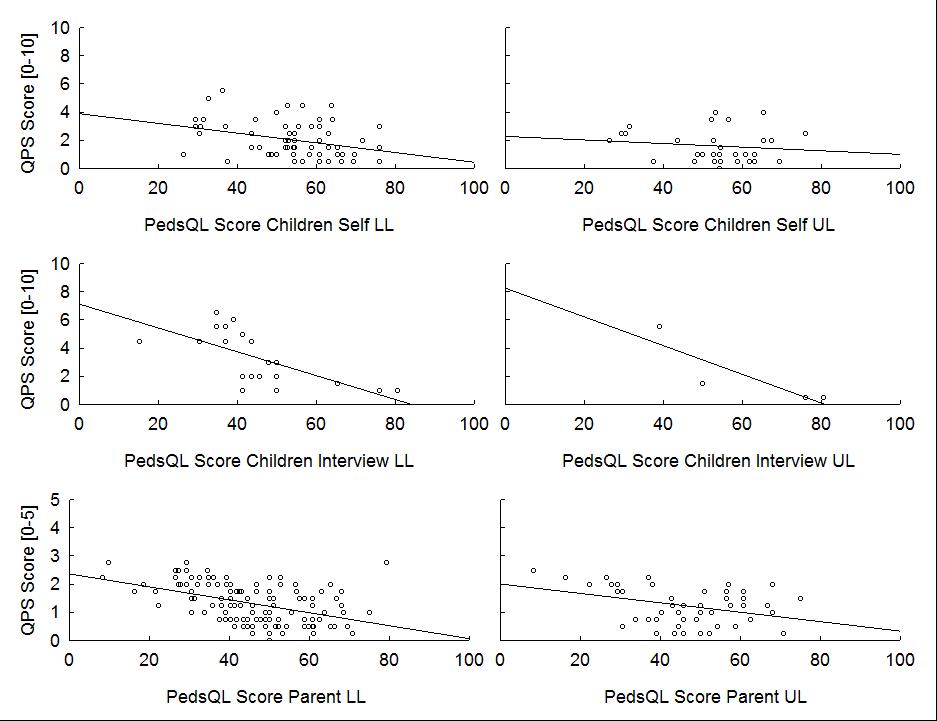

Supplement: Supplementary file 2 — QPS score relationship to PedsQL™, PedsQL™, Pediatric Quality of Life Inventory. (JPEG 73 kb) [file 12955_2017_804_MOESM2_ESM.jpg]
