# Supplementary material for: Initial psychometric validation of the questionnaire on pain caused by spasticity (QPS)
Source: Health Qual Life Outcomes. 2017 Nov 28;15:229. doi: 10.1186/s12955-017-0804-8 (PMC5704623; doi:10.1186/s12955-017-0804-8)
Supplement: Supplementary file 3 — Ordinal logistic regression analysis model of variables influencing QPS scores at baseline visit (V2). (DOCX 13 kb) [file 12955_2017_804_MOESM3_ESM.docx]

Additional file 3: Table S1 Ordinal logistic regression analysis model of variables influencing QPS scores at baseline visit (V2)

| **Patient characteristic** | **Child LL**  **(n = 106)** | | **Parent LL**  **(n = 120)** | |
| --- | --- | --- | --- | --- |
|  | Coefficient | t-statistic | Coefficient | t-statistic |
| Intercept | 26.1 | **3.409**** | 33.1 | **4.420**** |
| Age | –1.3 | **–3.254**** | –0.5 | –1.337 |
| Child gender | –2.7 | –0.978 | –4.6 | –1.725 |
| GMFCS | 2.9 | **2.732**** | 3.1 | **3.017**** |
| MACS | –1.5 | –1.079 | –0.4 | –0.275 |
| Parent overall health (of child) | –1.9 | –1.542 | –3.3 | **–2.721**** |
| Current child overall health | 3.7 | 1.869 | 1.2 | 0.575 |
| Use of assistive device^1^ | –7.4 | **–2.506*** | –7.5 | **–2.646**** |
| Therapy/medication^2^ | 15.4 | **4.692**** | 14.7 | **4.597**** |
| Overall model | F = 13.289**  R^2^ = 0.523  Adj R^2^ = 0.484 | | F = 13.561**  R^2^ = 0.494  Adj R^2^ = 0.458 | |

^1^Use of assistive device: 1 = use; 0 = don’t use.

^2^Therapy/medication: 1 = have; 0 = don’t have.

Significant associations are shown in bold, **p <*0.05; ***p <*0.01; ****p <*0.001.

GMFCS, Gross Motor Function Classification System; LL, lower limb; MACS, Manual Ability Classification System; UL, upper limb.
